# Frontiers in Global Women's Health: Choose to Challenge

**DOI:** 10.3389/fgwh.2021.676178

**Published:** 2021-05-24

**Authors:** Stephen Kennedy

**Affiliations:** Nuffield Department of Women's and Reproductive Health, University of Oxford, Oxford, United Kingdom

**Keywords:** women's health, maternal health, contraception, quality of life, women's mental health, sex and gender differences

**As a journal dedicated to improving the health and well-being of women across the world, the editorial team at** ***Frontiers in Global Women's Health*** **definitely #ChooseToChallenge on** **International Women's Day**.

*Frontiers in Global Women's Health* was launched as a new journal 1 year ago. Its mission was to “incorporate research from all disciplines related to the health problems facing women around the world.” We wanted to move away from the traditional biomedical model, which defines women's health purely in maternal, sexual and reproductive terms, toward a “life course approach that takes into account stressors as varied as climate change, infectious diseases, poverty, domestic violence and discrimination on the basis of gender.”

As the first health journal in the *Frontiers* series with the word *Global* in its title, we also aimed to stress the importance of addressing issues that affect vulnerable women in low-to-middle income countries (LMICs), particularly those experiencing large-scale demographic, epidemiological, socioeconomic, and environmental transitions, in keeping with the UN Sustainable Development Goals (SDGs), and the UN Global Strategy for Women's, Children's and Adolescents' Health (2016–2030).

Our first year has unquestionably been a resounding success. We have surpassed all our expectations thanks to the hard work of our five Specialty Section Chief Editors (Georgina Jones, *Quality of Life*; Jayashri Kulkarni, *Women's Mental Health*; Laura A Magee, *Maternal Health*; Chelsea Morroni, *Contraception* & *Family Planning*, and Sanne Peters, *Sex* & *Gender Differences in Disease*), as well as our 50 Associate Editors, 52 Guest Associate Editors, and 189 Review Editors. To have recruited such a large body of dedicated collaborators so rapidly is a testament to the need for such a journal. We must also not forget the remarkable *Frontiers* admin team, all of whom have been totally committed to the journal's mission from the outset.

What do I mean by success? To date, we have received a total of 141 manuscripts from authors in 42 countries, of which 43 have been accepted, 37 rejected, and the remainder are still under review. The published articles—a mixture of original research, reviews and study protocols—have been viewed and downloaded over 126,000 times.

Coincidentally, the COVID-19 pandemic started at roughly the same time that we launched the journal. We therefore devoted one of our first Research Topics to the effect of the pandemic on global women's health. Understandably, this one topic has so far attracted the most submissions. One article Moms Are Not OK: COVID-19 and Maternal Mental Health by Margie Davenport and colleagues already has an Attention Score of 457, putting it in the top 5% of all research outputs scored by Altmetric, including an article in the *The New York Times*. Given that the pandemic is continuing we decided last month to host a second edition of the COVID-19 & Women's Health Research Topic as the issues we now face from the pandemic are different to those we focused on last year: for example, new variants, vaccination (especially in pregnancy), long COVID, anxiety about fertility, and long-term psychosocial effects.

We have so far initiated a total of 17 Research Topics in a wide range of subjects from Maternal Health in Conflict Settings to What Works to Address Sex and Gender Disparities in Health and Disease? Our plan now is to build on the first year's success by starting at least one new Speciality Section devoted to Infectious Disease and encouraging suggestions for more Research Topics. In particular, we want to reach out to researchers in the social sciences and humanities to enable us to broaden the journal's scope even further. We are actively seeking submissions from a range of disciplines that can help to contextualize the significance of global women's health issues at individual and population levels. We aim to highlight the multitude of factors that affect women's health across the world and, perhaps more importantly, the impact that the failure to prioritize global women's health has had, is having and will have on society as a whole. In doing so, we want to attract as broad a readership as possible, which definitely means taking account of how these problems have been addressed in the non-biomedical literature. In other words, we will be seeking articles in the future that enrich the reader's understanding of the complexity of the issues facing clinicians, human rights lawyers, non-governmental organizations, funding agencies, and governments that wish to improve the health and well-being of women in both LMICs and high income countries. To the best of my knowledge no other journal seeks to incorporate such a rich variety of knowledge, expertise, and opinions pertaining specifically to global women's health.

Let me give you some examples of what I mean. It is difficult to appreciate global women's health issues without an understanding of their social and historical context, which is why we are encouraging submissions that take heed of Hegel's observation “The only thing we learn from history is that we learn nothing from history.” The present pandemic is a case in point. It is now acknowledged that Covid-19 is associated with substantially higher maternal and neonatal mortality and morbidity than was generally recognized at the start of the pandemic (1). Perhaps the lessons learned during the “Spanish flu” pandemic, which came in four waves between 1918 and 1920, resulting in the deaths of 20–50 million people worldwide, could have had a greater influence on thinking and health policies at the start of the present pandemic.

Then, the risks of prematurity and stillbirth were increased in affected women and, compared to non-pregnant women, those who were pregnant had a 50% higher chance of dying from respiratory complications (2). Interestingly, however, the “Spanish flu” pandemic permanently changed women's position in society because young men were so disproportionately affected. The worker shortage brought about by a combination of the First World War and the pandemic resulted in unprecedented numbers of women entering the workforce, particularly in roles that had traditionally been exclusively male (3). These changes contributed to the drive for greater women's rights through the suffragette movement. Contrast that with the evidence, highlighted in this journal in a number of articles, of the disproportionate effect of the Covid-19 pandemic on women's physical and mental well-being, and socio-economic position in society. The pandemic has also affected women's position in the labor market as most of the low-paid retail jobs and hospitality sector roles are undertaken by women and it is these roles that have seen the largest job losses.

Perhaps the most striking example of the pressing need for an interdisciplinary approach to global women's health relates to the gender-based health disparities associated with the effects of climate change, such as food and water insecurity, poor air quality, civil conflict, extreme weather events, and spread of infectious diseases (4, 5). Thus, we welcome submissions that address how climate change has led to greater risks of disease, malnutrition, sexual violence, poor mental health, lack of reproductive control, poor obstetric outcomes, and even death for women in many regions of the world. As others have highlighted (5), the disproportionate impact of climate change, especially on vulnerable women in LMICs, is mediated through socioeconomic and cultural, as well as physiological factors. An integrated approach is therefore required to find novel solutions that impact on policies and programmes so as to break down traditional barriers. We believe this journal has the potential to make a major contribution to those much needed efforts.

Our new Research Topic Economic Evaluation in Women's Health, led by the health economists Anju Devianee Keetharuth and Giulia Greco, is the latest demonstration of our commitment to expanding the scope of the journal. Their specific aim is to address the methodological challenges associated with assessing the wider social and economic benefits of investing in global women's health, particularly in LMICs. Although health economic analysis has become familiar to UK clinicians through the work of the National Institute for Health and Care Excellence (NICE), many in other countries may be unaware of the benefits, especially at population level, of evaluating the economics of interventions. Submissions in this and related fields are, therefore, welcomed.

Of course, the arts can also have a major impact on global women's health through the depiction of the human form and behaviors. For example, female readers of one of the top-selling fiction series of all time, the Fifty *Shades* novels that depict violence against women, have been shown to be at increased risk themselves of eating disorders and of being verbally and emotionally abused (6). On a lighter note, many people will never have seen Damien Hirst's The Miraculous Journey−14 giant uterine sculptures in bronze depicting a fetus' journey from conception to birth, which are located outside the new Sidra Mother and Child Hospital in Qatar. So, we plan to attract submissions related to the arts that enrich the mind and broaden the reader's appreciation of global women's health.

In summary, much has been achieved in a relatively short space of time. However, we are an ambitious group of editors who would like, in time, *Frontiers in Global Women's Health* to be the single most influential journal in the field, encompassing every aspect pertaining to the promotion of improved health and well-being for all women of all ages throughout the world. The need for such a journal is paramount given the very real possibility that the COVID-19 pandemic has disrupted health care provision to such an extent globally that decades of past improvements are at risk of being reversed.

## Author Contributions

The author confirms being the sole contributor of this work and has approved it for publication.

## Conflict of Interest

The author declares that the research was conducted in the absence of any commercial or financial relationships that could be construed as a potential conflict of interest.
